# Matrix metalloproteinases mediate influenza A-associated shedding of the alveolar epithelial glycocalyx

**DOI:** 10.1371/journal.pone.0308648

**Published:** 2024-09-23

**Authors:** Kaitlyn R. Schaaf, Stuart R. Landstreet, Nathan D. Putz, Samantha K. Gonski, Jason Lin, Charity J. Buggs, Dustin Gibson, Christophe J. Langouët-Astrié, Christopher S. Jetter, Nicolas M. Negretti, Jennifer M. S. Sucre, Eric P. Schmidt, Lorraine B. Ware, Julie A. Bastarache, Ciara M. Shaver

**Affiliations:** 1 Vanderbilt University, Nashville, Tennessee, United States of America; 2 Department of Pathology, Microbiology, and Immunology, Vanderbilt University Medical Center, Nashville, Tennessee, United States of America; 3 Division of Allergy, Pulmonary, and Critical Care Medicine, Department of Medicine, Vanderbilt University Medical Center, Nashville, Tennessee, United States of America; 4 Division of Pulmonary and Critical Care, Department of Medicine, University of Colorado Anschutz, Denver, Colorado, United States of America; 5 Department of Neonatology, Monroe Caroll Children’s Hospital at Vanderbilt, Nashville, Tennessee, United States of America; 6 Division of Pulmonary and Critical Care, Department of Medicine, Massachusetts General Hospital, Boston, Massachusetts, United States of America; 7 Department of Cell and Development Biology, Vanderbilt University, Nashville, Tennessee, United States of America; Gifu University School of Medicine Graduate School of Medicine: Gifu Daigaku Igakubu Daigakuin Igakukei Kenkyuka, JAPAN

## Abstract

**Background:**

The alveolar epithelium is protected by a heparan sulfate-rich, glycosaminoglycan layer called the epithelial glycocalyx. It is cleaved in patients with acute respiratory distress syndrome (ARDS) and in murine models of influenza A (IAV) infection, shedding fragments into the airspace from the cell surface. Glycocalyx shedding results in increased permeability of the alveolar-capillary barrier, amplifying acute lung injury. The mechanisms underlying alveolar epithelial glycocalyx shedding in IAV infection are unknown. We hypothesized that induction of host sheddases such as matrix metalloproteinases (MMPs) during IAV infection results in glycocalyx shedding and increased lung injury.

**Materials and methods:**

We measured glycocalyx shedding and lung injury during IAV infection with and without treatment with the pan-MMP inhibitor Ilomastat (ILO) and in an MMP-7 knock out (MMP-7KO) mouse. C57BL/6 or MMP-7KO male and female mice were given IAV A/PR/8/34 (H1N1) at 30,000 PFU/mouse or PBS intratracheally. For some experiments, C56BL/6 mice were infected in the presence of ILO (100mg/kg) or vehicle given daily by IP injection. Bronchoalveolar lavage (BAL) and lung tissue were collected on day 1, 3, and 7 for analysis of glycocalyx shedding (BAL Syndecan-1) and lung injury (histology, BAL protein, BAL cytokines, BAL immune cell infiltrates, BAL RAGE). Expression and localization of the sheddase MMP-7 and its inhibitor TIMP-1 was examined by RNAScope. For in vitro experiments, MLE-12 mouse lung epithelial cells were cultured and treated with active or heat-inactivated heparinase (2.5 U/mL) prior to infection with IAV (MOI 1) and viral load and MMP-7 and TIMP-1 expression analyzed.

**Results:**

IAV infection caused shedding of the epithelial glycocalyx into the BAL. Inhibition of MMPs with ILO reduced glycocalyx shedding by 36% (p = 0.0051) and reduced lung epithelial injury by 40% (p = 0.0404). ILO also reduced viral load by 68% (p = 0.027), despite having no significant effect on lung cytokine production. Both MMP-7 and its inhibitor TIMP-1 were upregulated in IAV infected mice: MMP-7 colocalized with IAV, while TIMP-1 was limited to cells adjacent to infection. However, MMP-7KO mice had similar glycocalyx shedding, epithelial injury, and viral load compared to WT littermates, suggesting redundancy in MMP sheddase function in the lung. In vitro, heparinase treatment before infection led to a 52% increase in viral load (p = 0.0038) without altering MMP-7 or TIMP-1 protein levels.

**Conclusions:**

Glycocalyx shedding and MMPs play key roles in IAV-induced epithelial injury, with significant impact on IAV viral load. Further studies are needed to understand which specific MMPs regulate lung epithelial glycocalyx shedding.

## Introduction

Influenza A virus (IAV) is one of the most common human respiratory infections and is a significant source of morbidity and mortality, with approximately 550,000 hospitalizations and 35,000 deaths in the United States in a typical endemic season [1]. Patients with severe IAV often develop acute respiratory distress syndrome (ARDS) with pulmonary edema and respiratory failure. Despite extensive critical care support, many of these patients die. Autopsies from patients who succumb to IAV show findings consistent with ARDS, including intense alveolar inflammation and destruction of the alveolar-capillary barrier [2]. The molecular mechanisms that lead to acute lung injury in the setting of IAV infection are not well understood.

The alveolar glycocalyx, a heparan sulfate-rich, glycosaminoglycan layer on the luminal surface of the alveolar epithelium [3], has a critical role in maintaining lung health and, when damaged, may contribute to the pathogenesis of ARDS [4]. In healthy lungs, the alveolar epithelial glycocalyx helps maintain alveolar fluid balance, aids in surfactant maturation, and controls alveolar barrier permeability [5]. Studies in mice show that degradation of the alveolar glycocalyx by direct intratracheal (IT) administration of heparinase is sufficient to cause alveolar barrier permeability [6]. Glycocalyx shedding is a feature of several other murine models of direct lung injury, including IT lipopolysaccharide (LPS) [6], bleomycin [7, 8], MRSA [9] and IAV [10], offering a potential common mechanism by which damage to the lung epithelium leads to increased barrier permeability. Glycocalyx shedding also occurs in humans with ARDS, where the amount of heparan sulfate in the airspace positively correlates with biomarkers of lung injury [11]. These studies indicate that the alveolar epithelial glycocalyx plays an important role in maintaining the healthy alveolar microenvironment and that loss of the glycocalyx is detrimental to lung function.

Sheddases are membrane bound enzymes that cleave extracellular portions of proteins and glycoproteins and contribute to glycocalyx shedding. Multiple matrix metalloproteases (MMPs) have been shown to have sheddase activity [12]. While MMPs have many roles in the lung including modulating epithelial repair and normal epithelial development [13], several studies support the role of MMPs in inflammation and acute lung injury [14]. MMP-2, -7, -9, and -14 have been implicated in lung injury models ranging from IT bleomycin to *Pseudomonas aeruginosa* infection [6, 8, 15–18]. Several inhibitors of MMPs, including tissue inhibitor of metalloproteinase (TIMP)-1 and TIMP-2 may also be induced during acute lung injury [15, 19, 20]. The balance of these counter regulatory TIMPs in relation to MMPs can impact the overall effect of MMPs during acute lung injury. During IAV, whether MMPs drive alveolar epithelial glycocalyx shedding and viral progression remains unknown.

In this study, we tested the hypothesis that IAV-induced glycocalyx shedding occurs through upregulation of the sheddase MMP-7 and contributes to more severe IAV infection and viral load.

## Materials and methods

### Mouse strains

Male and female, wild-type (WT) C57BL/6 mice were purchased from Jackson Laboratories at 8 weeks old and allowed to acclimatize to our facility for 1 week before experimentation. B6.129-*Mmp7*^*tm1Lmm*^/J mice (MMP-7KO, strain number 005111) heterozygotes were cryo-recovered from Jackson Laboratories, then bred in our animal facility to generate MMP-7KO and WT littermates. Genotype was confirmed using standard PCR procedure from Jackson Laboratory. Male and female mice homozygous for MMP-7KO, and their littermate controls, were used in experiments at 8- to 16-weeks of age. All studies were carried out in strict accordance with the recommendations in the Guide for the Care and Use of Laboratory Animals of the National Institutes of Health. All protocols were approved by the Institutional Animal Care and Use Committee of Vanderbilt University Medical Center (Protocol Number: M1600006-02). All personnel preforming experiments were fully trained and compliant with protocol requirements.

### Murine model of influenza pneumonia

Mice were anesthetized with 3.5% isoflurane and administered 30,000 PFUs of mouse adapted H1N1 A/Puerto Rico/8/32 (PR8) [21] in 30 μL or PBS control intranasally in alternating nostrils while held supine at a 45^◦^ angle [22, 23]. To ensure viral solution fully entered the lungs, all mice were administered an additional 30 μL of PBS in the same manner. Mice were returned to cages prone and allowed to right themselves naturally. Mice were monitored for weight loss daily [22, 23] until day 7 post infection when animals were euthanized by Euthasol (pentobarbital sodium and phenytoin solution) injection, followed by exsanguination. Animals with >20% weight loss were euthanized as above immediately upon discovery as a humane endpoint, as required by our animal protocol. For some experiments, the final samples were obtained at day 6 after infection to avoid bias from animal mortality. No animal death occurred before meeting the criteria for euthanasia. For select experiments, the pan-MMP inhibitor Ilomastat (ILO; SelleckChem) was diluted in solubility buffer (2% DMSO, 2% Tween-80, 40% PEG300 v/v in distilled H_2_O) and given at 100 mg/kg by intraperitoneal injection at the time of infection, then once daily thereafter. Equal volume of solubility buffer was used as vehicle control. For all experiments, every effort was made to minimize suffering, including providing access to DietGel 76A nutrition and hydration supplement from day 3 of infection onwards. For each individual experiment, 2–3 control animals and 3–6 infected animals were used in each treatment group at each timepoint. All experiments were performed on at least two separate days. In total, 249 animals were used in this study.

### Sample collection and lung injury assessment

Bronchoalveolar lavage (BAL) and lung tissue samples were collected at day 1, 3, and 6 or 7 after infection. At each timepoint, BAL was performed with 0.7 mL PBS. Lungs were flash frozen and stored at -80°C until analysis. For experiments with histologic analysis, no BAL was performed and the lungs were fixed as outlined below. Inflammatory cells in BAL were manually enumerated and differentials determined after DiffQuik staining as previously described [24–26]. Alveolar-capillary barrier function was assessed by measurement of BAL protein by BCA assay. Glycocalyx shedding was measured by Syndecan-1 ELISA on BAL (Novus Biologicals, Centennial, CO). BAL cytokines were measured by multiplex electro-chemiluminescent immunoassay (Meso Scale Diagnostics, Gaithersburg, MD), except for Interferon-α which was measured by ELISA (Abcam, Waltham, MA). Flash-frozen lung tissue was homogenized and RNA extracted by RNeasy protocol (Qiagen, Germantown, MD), cDNA was synthesized by VILO Superscript (Biorad, Hercules, CA), and viral load determined by qPCR targeting segment 8 of PR8 genome (Forward primer: CGGTCCAAATTCCTGCTGA, Reverse primer: CATTGGGTTCCTTCCATCCA). Similarly, expression of MMP-7, TIMP-1, MMP-2, MMP-9, and TIMP-2 was quantified by qPCR using Taqman primers. For examination of MMP-7 and TIMP-1 protein, flash-frozen lung tissue was homogenized and lysed with RIPA buffer with protease and phosphatase inhibitors and MMP-7 and TIMP-1 proteins measured by ELISA (Novus Biologicals, Centennial, CO). For all ELISAs, samples below the limit of detection were replaced with a value equal to half of the lowest standard.

### Histological analysis

Lungs were perfused with 4% paraformaldehyde, embedded in paraffin, sectioned, and mounted on slides, then stained with hematoxylin and eosin. Lung injury was quantified by a researcher blinded to experimental groups as previously described [24, 26]. Briefly, 10 nonoverlapping high-powered fields (20×) were manually assessed for septal thickening, inflammation, edema, and hemorrhage on a scale of 0–5 for each criterion (with 0 meaning normal and 5 meaning most severe injury). Each component score was averaged across the 10 fields, then summed to get the total lung injury score for each animal.

### In vitro cell treatment and IAV infection

MLE-12 murine lung epithelial cells were grown in HITES media (DMEM/F12 1:1 with 2% fetal bovine serum (FBS), 0.005mg/mL insulin, 0.01mg/mL transferrin, 30nM sodium selenite, 10nM hydrocortisone, 10nMm β-estradiol, 10mM HEPES, and 2mM L-glutamine) according to America Type Culture Collection recommendations. Cells were plated 48h before use in experiments to allow a completely confluent cell monolayer to form and to promote establishment of the glycocalyx. To test the effect of glycocalyx degradation on IAV infection in epithelial cells, MLE-12 cells were treated with 2.5 units of active or heat-inactivated (by incubating at 100°C for 15min) heparinase I/III blend from *Flavobacterium heparinum* (Sigma-Aldrich, St. Louis, MO) for 6 hours. Then, MLE-12 cells were infected with IAV or PBS control at a multiplicity of infection (MOI) of 1 in the minimum possible volume (300 μL per well of 24-well plate) of HITES media without FBS. After 1 hour of viral attachment, complete culture media was added for 24 hours. The cell monolayer was lysed and RNA extracted, cDNA generated, and viral load determined by RT-qPCR as described above. Similar experiments were conducted and cell monolayers lysed with RIPA buffer with protease and phosphatase inhibitors to collect total cell protein content for MMP-7 and TIMP-1 quantification by ELISA. Cytokine secretion into the supernatant was measured by multiplex electro-chemiluminescent immunoassay (Meso Scale Diagnostics, Gaithersburg, MD), except for Interferon-α which was measured by ELISA (Abcam, Waltham, MA).

### RNA in situ hybridization

Lungs were probed using RNAScope (ACDBio, Newark, CA) according to manufacturer’s instructions and previous publications [27, 28]. In brief, unstained sections from paraffin-embedded lungs were deparaffinized, and probed with MMP-7, TIMP-1, MMP-2, MMP-9, or TIMP-2 as the channel 1 probe, and with IAV as the channel 2 probe. Channel 1 was visualized with Cyanine3, channel 2 with Cyanine5, and DAPI used as a nuclear counter stain to visualize cells. Positive control probe (PPIB) and negative control probe (DapB) were purchased from the company. Images were captured using the BZ-X800 microscope (Keyence, Itasca, IL) equipped with appropriate filters. Lungs were evaluated by imaging at least three separate regions of IAV signal, then visually examining the abundance and localization of the MMP or TIMP signal. Excitation and exposure settings were kept consistent for each channel within each treatment day.

### Statistical analyses

Statistical analysis was done in GraphPad Prism version 10 and specific statistical tests for each analysis are specified within figure legends. Mann-Whitney U test was used to compare continuous variables across two groups. Two-way ANOVA was used to compare across groups with two independent variables with Tukey’s post-hoc test to define specific relationships. Post hoc statistical analysis of significant phenotypes was performed to assess for sex-specific differences. All data are shown as median with error bars representing the interquartile range unless otherwise specified.

## Results

### The alveolar epithelial glycocalyx is shed during IAV infection

**Fig 1:**
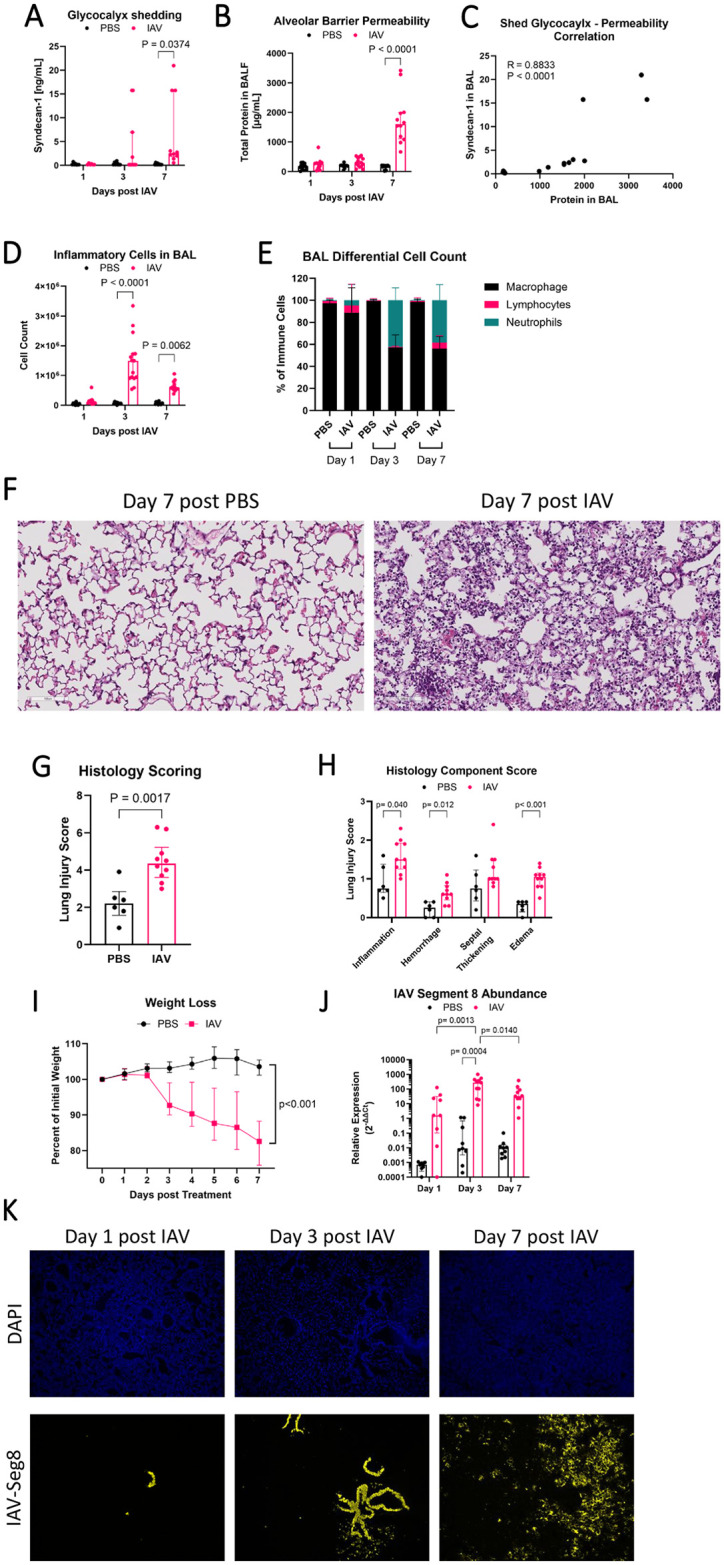
IAV causes alveolar glycocalyx shedding during acute infection in mice. Wild-type C57BL/6 mice were intranasally administered PR8 influenza or PBS. On day 7, IAV infected mice had (A) increased shedding of the alveolar epithelial glycocalyx as measured by glycocalyx anchoring protein Syndecan-1 in bronchoalveolar lavage (BAL) fluid (n = 6–11 [7 male, 4 female]; overall p = 0.015 for infection, two-way ANOVA with significant post-hoc relationships within days indicated) and (B) increased alveolar-capillary barrier permeability as measured by total BAL protein (n = 10–14 [10 male, 4 female], overall p<0.0001 for infection, overall p<0.0001 for time, two-way ANOVA with significant post-hoc relationships within days indicated). (C) Glycocalyx shedding correlates with alveolar barrier permeability (n = 16 [12 male, 4 female], Spearman’s Correlation). IAV infected mice also have (D) increased total inflammatory cells in BAL (n = 10–15 [11 male, 4 female], overall p<0.0001 for infection, overall p<0.0001 for time, two-way ANOVA with significant post-hoc relationships within days indicated), with (E) increased neutrophil influx at day 3 and day 7 (P<0.001 for each, Mann-Whitney U for each day with Holm-Sidak correction). (F) Representative histologic images (hematoxylin and eosin) from control and IAV infected lungs at day 7 are shown and (G) lung injury quantification by assessment of ten non-overlapping visual fields using a validated lung injury score indicates significant lung injury in IAV mice (n = 6–10 [5 male, 5 female], Mann-Whitney U). (H) Components of the lung injury score show that inflammation, hemorrhage, and edema are the primary manifestations of IAV-induced lung injury (Mann-Whitney U for each category with Holm-Sidak correction). (I) IAV mice have increased weight loss beginning at day 3 (n = 9–42 [22 male, 20 female]; mixed model ANOVA). (J) IAV viral load peaks at day 3, as measured by relative expression (ΔΔCt) analysis to IAV day 1 of RT-qPCR of IAV genome segment 8 (n = 8–11 [9 male, 4 female], overall p = 0.001 for infection and overall p = 0.012 for time, two-way ANOVA with significant post-hoc relationships indicated). (K) IAV infection progresses from larger airways on day 1, to smaller airways on day 3, to alveoli on day 7. RNA in situ hybridization shows IAV genome segment 8 in yellow and DAPI nuclear counterstain in blue. There are no statistically significant sex-specific differences in these parameters.

To test whether alveolar epithelial glycocalyx shedding contributes to acute lung injury during IAV infection, we defined the time course of IAV infection in male and female wild-type C57BL/6 mice over 7 days with assessment of glycocalyx shedding and multiple parameters of lung injury. The glycocalyx core protein syndecan-1 was increased 16-fold in BAL at day 7 after IAV infection (Fig 1A), a time course consistent with previous reports of IAV-induced glycocalyx shedding [10]. At the same time point, alveolar-capillary barrier permeability increased 9-fold (Fig 1B; BAL protein median (interquartile range) for PBS: 179 μg/mL (71–209 μg/mL) and for IAV: 1601 μg/mL (1110–1997 μg/mL). There was a positive correlation between glycocalyx shedding and alveolar-capillary barrier permeability at day 7 (Fig 1C). BAL inflammatory cell counts were increased 25-fold at day 3 and 10-fold at day 7 in IAV-infected mice (Fig 1D) with increased neutrophils in particular (Fig 1E; 42% and 38% on day 3 and 7, respectively, in IAV compared to less than 1% in PBS). Histologic scoring confirmed lung injury at day 7 (Fig 1F and 1G) with statistically significant increases in inflammation (2-fold), edema (3-fold), and hemorrhage (2-fold; Fig 1H). Preceding maximal lung injury and glycocalyx shedding, IAV-infected mice had systemic illness with an average of 15% weight loss (Fig 1I). Quantification of viral burden showed that viral load increased 172-fold between day 1 and 3, with peak viral load on day 3 (Fig 1J). Spatial assessment of IAV locations showed that IAV genome is primarily confined to large airways on days 1 and 3 with dissemination into the alveoli occurring by day 7 (Fig 1K). Taken together, these data show that glycocalyx shedding correlates with lung injury during IAV pneumonia.

### Glycocalyx shedding is reduced by inhibiting matrix metalloproteases with Ilomastat

**Fig 2:**
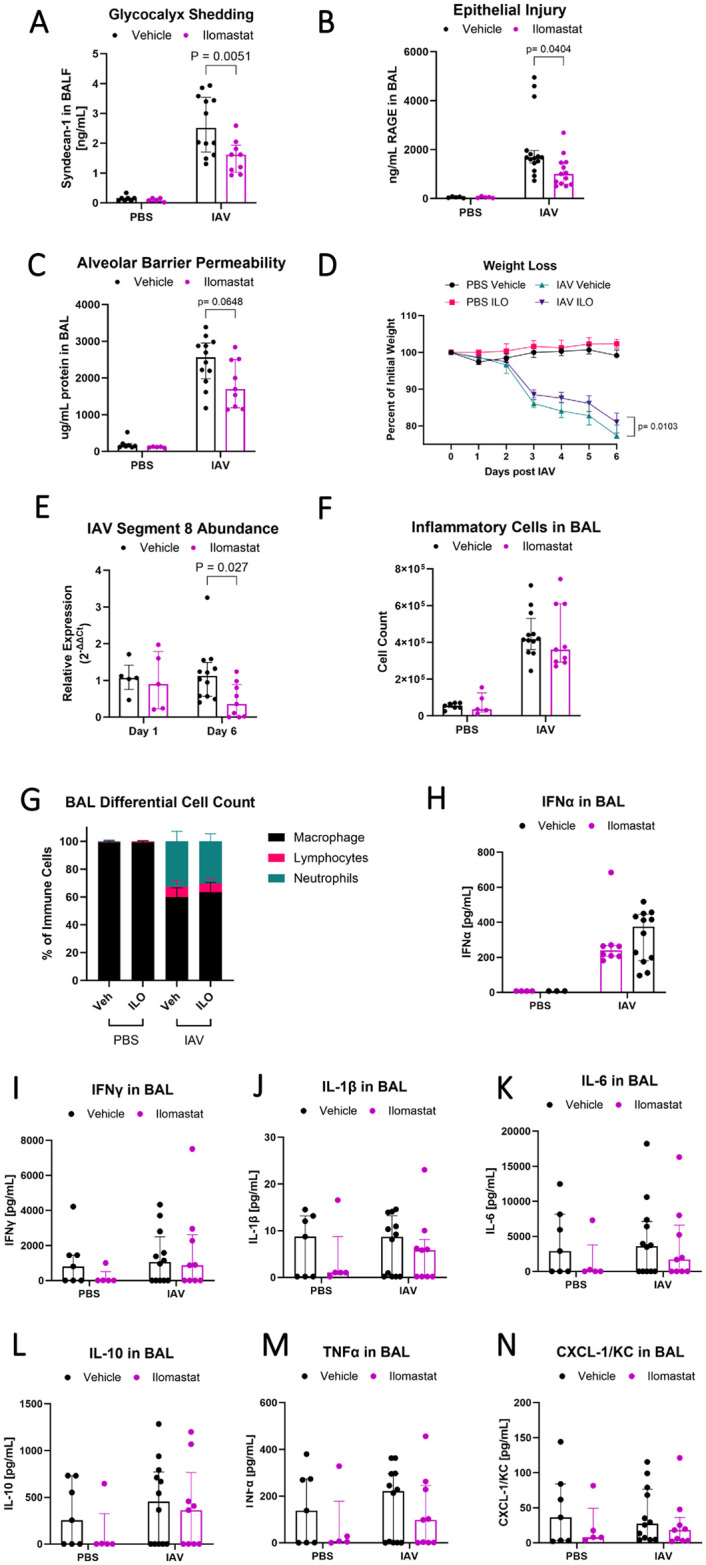
Pan-MMP inhibitor Ilomastat reduces glycocalyx shedding and epithelial injury from IAV. Wild-type C57BL/6 mice were instilled with PR8 or PBS in the presence or absence of 100mg/kg Ilomastat (ILO) or vehicle control in equal volume administered intraperitoneally daily from the time of infection. On day 6, ILO-treated, IAV-infected mice had (A) reduced glycocalyx shedding (n = 5–12 [all male], overall p<0.0001 for infection, overall p = 0.028 for drug treatment, two-way ANOVA with significant post-hoc relationships within infection groups indicated), (B) less epithelial damage as indicated by RAGE in the BAL (n = 5–12 [all male], overall p<0.0001 for infection, overall p = 0.0593 for drug treatment, two-way ANOVA with post-hoc relationships within infection groups indicated), and (C) numerically less alveolar-capillary permeability (n = 5–12 [all male], overall p<0.0001 for infection, overall p = 0.083 for drug treatment, two-way ANOVA with post-hoc relationships within infection groups indicated). ILO treated animals also experienced (D) less weight loss (n = 5–18 [all male], mixed-model ANOVA). (E) ILO treatment had no impact on day 1 viral load, but had reduced viral load on day 6 as seen by relative expression (ΔΔCt) analysis to same day vehicle control (n = 5–12 [all male], Mann-Whitney U test for each day with Holm-Sodak correction). On day 6, ILO treatment had no significant impact on (F) the number or (G) composition of BAL inflammatory cells or on (H-N) the concentration of several pro-inflammatory cytokines in BAL (n = 5–12 [all male], two-way ANOVA).

MMPs are able to cleave glycocalyx components during experimental acute lung injury, but the mechanisms of glycocalyx shedding during IAV infection are unknown. To test the mechanistic role of MMPs in glycocalyx shedding, we used the pan-MMP inhibitor Ilomastat (ILO). ILO has inhibitory activity against nine different MMPs, including MMP-2, MMP-7, and MMP-9. Mice received intraperitoneal ILO or vehicle daily during IAV infection, and samples were collected on day 6 to minimize bias from mortality. Pharmacologic inhibition of MMPs with ILO decreased epithelial glycocalyx shedding into the airspace by 36% (Fig 2A). ILO reduced lung epithelial injury by 40% as measured by BAL RAGE (Fig 2B) and showed a trend towards reduced damage to the alveolar-capillary barrier by 34% (Fig 2C, p = 0.0648). Treatment with ILO also attenuated weight loss (23% weight loss from baseline in vehicle, 19% in ILO; Fig 2D) compared to vehicle controls. MMP inhibition with ILO did not diminish establishment of IAV infection on day 1 but did decrease viral load at day 6 by 68% (Fig 2E). These ILO-associated changes in IAV viral dynamics were not explained by changes in inflammation, as the number and differential of BAL inflammatory cells and expression of pro-inflammatory cytokines in BAL did not significantly differ (Fig 2F–2N). These data suggest that MMPs are the key sheddases responsible for glycocalyx destruction during IAV infection and that sheddase activity occurs independently of the severity of lung inflammation.

### The effect of matrix metalloproteases on viral dynamics and glycocalyx injury is not due to differences in inflammatory responses

**Fig 3:**
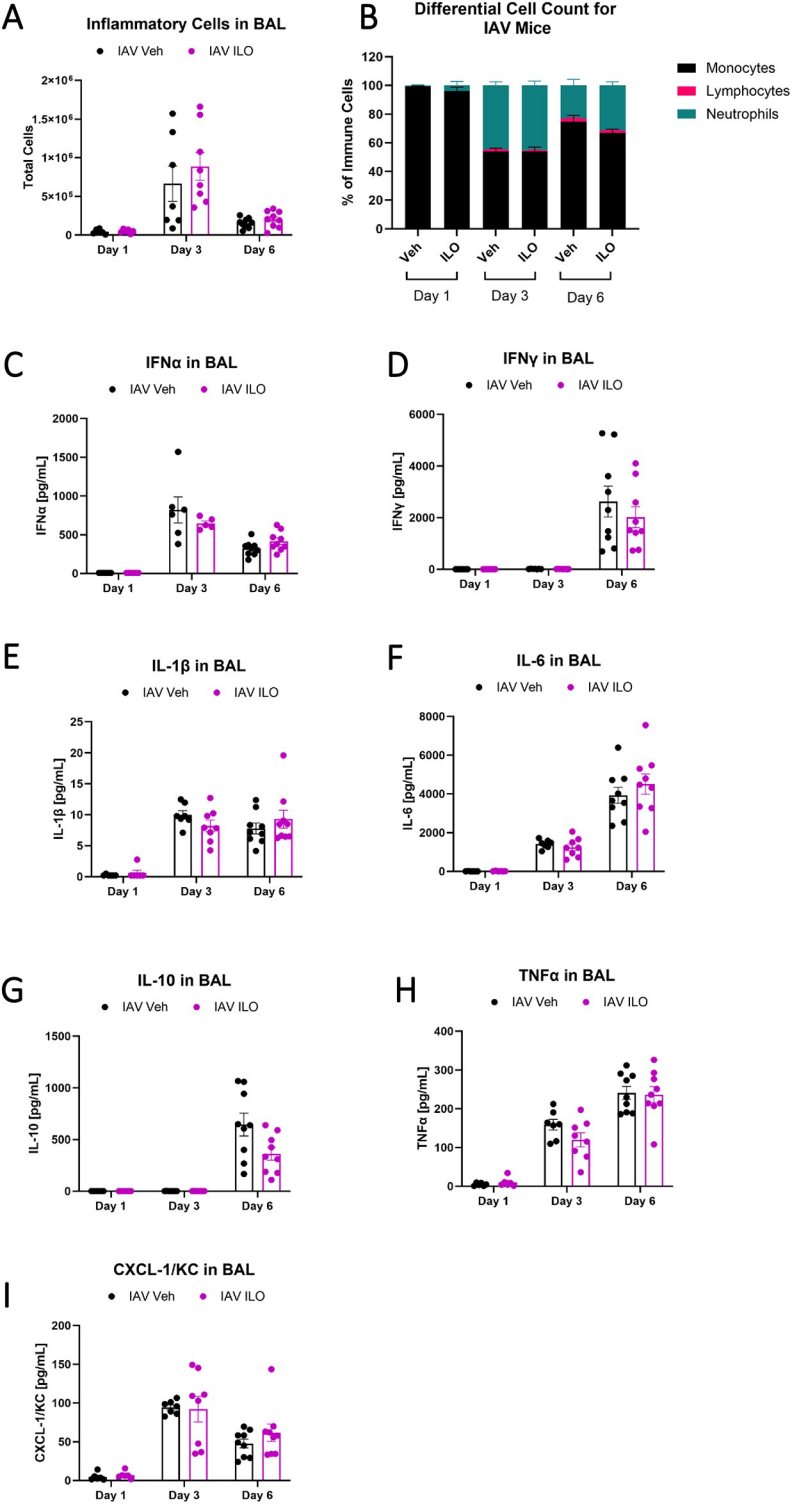
Ilomastat does not alter the inflammatory response to IAV. Male and female wild-type C57BL/6 mice were instilled with PR8 or PBS in the presence or absence of 100mg/kg ILO or vehicle control administered intra-peritoneally daily from the time of infection. Samples were collected at day 1, 3, and 6. ILO treatment did not alter the (A) number or (B) composition of immune cells in BAL and did not significantly alter the BAL concentration of (C) Interferon (IFN)-α, (D) IFN-γ, (E) Interleukin (IL)-1β, (F) IL-6, (G) IL-10, (H) TNF-α, or (I) CXCL1/KC during IAV (n = 6–9 [4 male, 5 female], two-way ANOVA). There are no statistically significant sex-specific differences in these parameters.

Because MMP inhibition resulted in reduced IAV viral load in the lung while reducing glycocalyx shedding and lung injury, we tested whether MMP inhibition augmented anti-viral inflammation prior to epithelial injury and glycocalyx shedding. We performed a time course experiment in which mice were treated with ILO or vehicle and then infected with IAV and samples collected at day 1, 3, and 6. There were no differences in BAL inflammatory cell influx, cell differentials, or BAL cytokines interferon (IFN)-α, IFN-γ, interleukin (IL)-1β, IL-6, IL-10, TNF-α, or CXCL-1/KC between ILO and vehicle treated mice (Fig 3). These data show that the impact of ILO on viral dynamics and parameters of lung injury was not explained by changes in earlier immune responses to IAV.

### Destruction of the epithelial glycocalyx increases viral load in vitro

**Fig 4:**
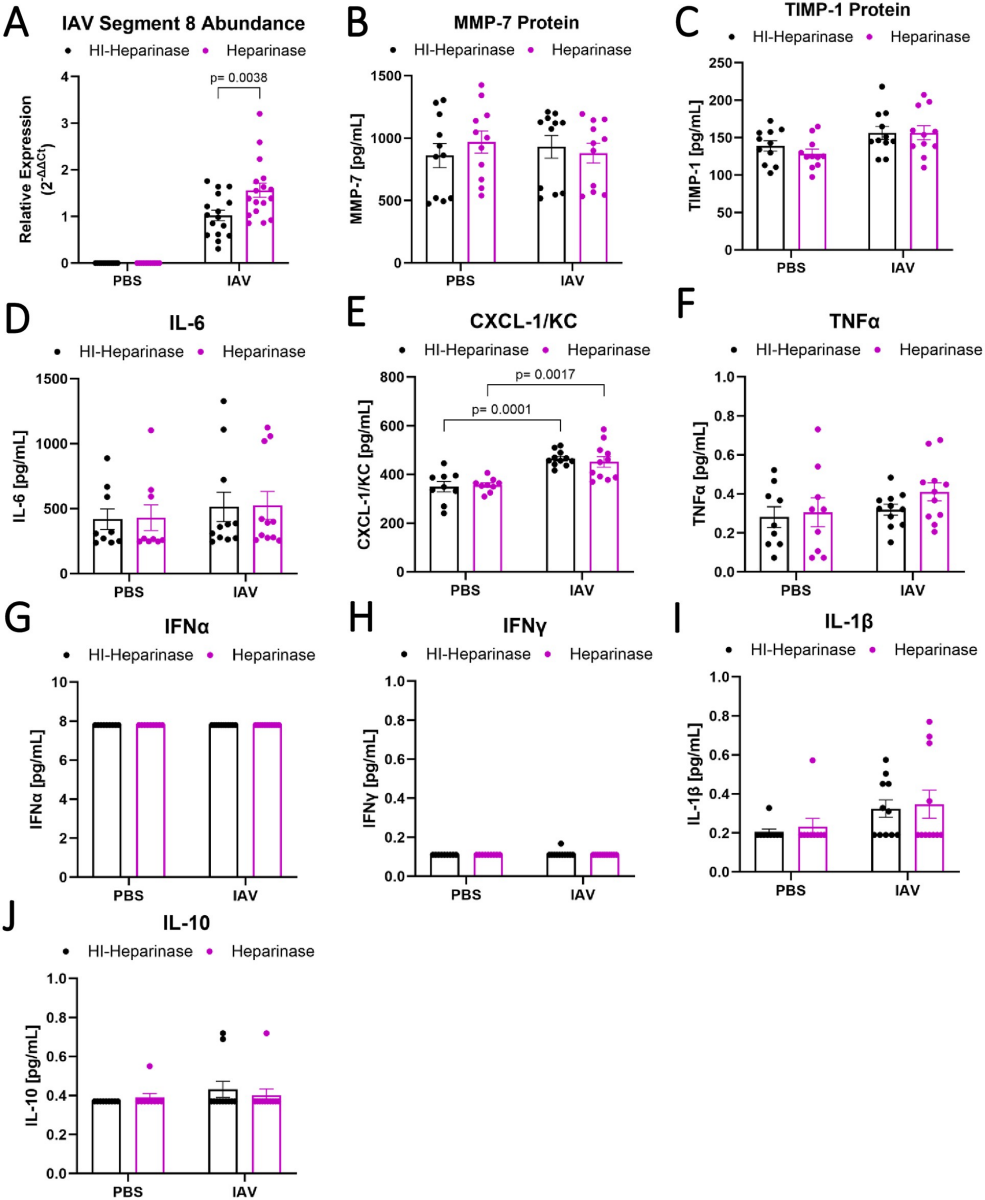
Destruction of the glycocalyx in epithelial cells increases IAV viral load. Confluent MLE-12 murine epithelial cells were treated with active or heat-inactivated heparinase I/III for 6h to shed the glycocalyx before infecting with PR8 at an MOI of 1 for 24 hours. (A) Destruction of the glycocalyx by heparinase I/III leads to increased viral load after PR8 infection (n = 12–17, overall p<0.0001 for infection, overall p = 0.025 for heparinase treatment, two-way ANOVA with significant post-hoc relationships between groups indicated). (B) MMP-7 and (C) TIMP-1 protein are not significantly altered by heparinase treatment (n = 11, two-way ANOVA), nor are (D-J) the concentration of several pro-inflammatory cytokines in the supernatant (n = 9–11, two-way ANOVA). For (G) IFNα, (H) IFNγ, (I) IL-1β, and (J) IL-10, samples are at or near the limit of detection.

Because we observed lower viral load in the lung when MMPs were inhibited, we sought to test for a direct role of glycocalyx shedding in viral propagation. To do this, MLE-12 murine lung epithelial cells were infected with IAV for 24 hours after treatment with either active heparinase to induce glycocalyx destruction or with heat-inactivated heparinase as a control. Destroying the glycocalyx with heparinase prior to IAV infection in vitro increased viral load 52% (Fig 4A). Heparinase treatment did not alter MMP-7 (Fig 4B) or TIMP-1 (Fig 4C) protein levels, nor did it affect IL-6, CXCL-1/KC, or TNFα production (Fig 4D–4F) in response to IAV. IFNα, IFNγ, IL-1β, and IL-10 were also measured and showed no appreciable signal in any treatment (Fig 4E–4J). Taken together, these data indicate that glycocalyx integrity is directly linked to viral load.

### MMP-7 and its inhibitor TIMP-1 have differential RNA expression and localization after IAV

**Fig 5:**
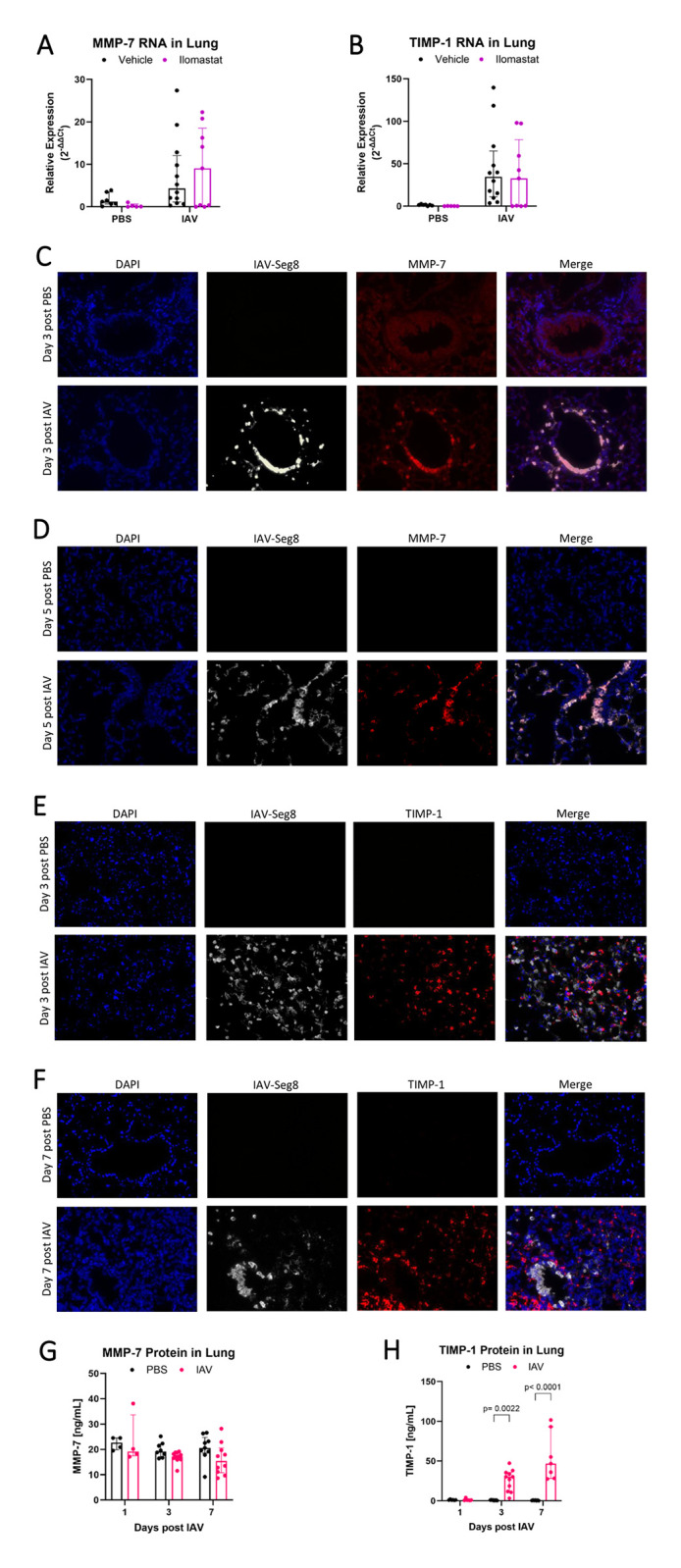
MMP-7 and its inhibitor TIMP-1 have differential RNA localization with IAV genome. On day 6 post infection, (A) MMP-7 and (B) TIMP-1 RNA expression in total lung lysates was increased by infection but not by ILO treatment (n = 5–12 [all male], MMP-7 overall p = 0.0086 for infection, TIMP-1 overall p = 0.0038 for infection, two-way ANOVAs). MMP-7 expression is limited to cells directly infected with IAV in (C) large, proximal airways at day 3 (n = 3 [all male], ≥9 infected fields per mouse) and in (D) smaller, distal airways at day 5 (n = 3–4 [all male], ≥9 infected fields per mouse). TIMP-1 expression is limited to cells adjacent but not directly infected with IAV at (E) day 3 (n = 4 [all male], ≥6 infected fields per mouse) and (F) day 7 (n = 4 [all male], ≥6 infected fields per mouse). IAV genome segment 8 and MMP-7 or TIMP-1 localization in whole lung tissue slices is visualized by in situ RNA hybridization, with 40x image fields representative of IAV and PBS mice shown. (G) There is no significant difference in MMP-7 protein in whole lung homogenates during IAV infection (n = 4–11 [7 male, 4 female], two-way ANOVA). (H) TIMP-1 total protein in whole lung homogenates increases on day 3 and day 7 after IAV (n = 6–11 [7 male, 4 female], overall p<0.0001 for infection, overall p<0.0001 for time, two-way ANOVA with significant post-hoc relationships within days indicated).

We hypothesized that IAV infection of lung epithelium would lead to upregulation of MMPs that would then cleave the alveolar glycocalyx and exacerbate lung injury. Next, to identify the specific MMPs that may explain the impact of Ilomastat on glycocalyx shedding during IAV, we examined the expression of several MMPs and their native regulators (TIMPs) during IAV infection. At the whole lung level, there were no consistent increases in gene expression of MMP-2, MMP-9, or their inhibitor TIMP-2 during IAV (S1 Fig). For MMP-7 and its inhibitor TIMP-1, there was a significant increase in whole lung RNA in infected animals at day 6, approximately 5-fold and 30-fold respectively, with no changes due to ILO treatment (Fig 5A and 5B). However, because IAV infection can be patchy, we next used RNA in situ hybridization (RNAScope) to visualize the spatiotemporal relationship between the IAV genome and MMP-7 or TIMP-1 RNA. MMP-7 expression was only detected in IAV infected cells, in proximal airways at day 3 (Fig 5C) and in more distal airways at day 5 (Fig 5D). In contrast, TIMP-1 did not co-localize with IAV, as TIMP-1 was only present in cells adjacent to IAV-infected cells (Fig 5E and 5F). Additional lower magnification images are provided in S2 Fig. RNAScope also confirmed that MMP-2, MMP-9, and TIMP-2 expression did not vary during IAV infection (S3 Fig). At the protein level in whole lung, MMP-7 was not increased during IAV infection (Fig 5G), whereas TIMP-1 was significantly increased at day 3 and day 7 (Fig 5H). Collectively, these data demonstrate that MMP-7 and TIMP-1 are the sheddase system most upregulated during IAV infection and offer further information about the spatial nature of their relationship with IAV.

### MMP-7 is not required for glycocalyx shedding, barrier permeability, or inflammation during IAV infection

**Fig 6:**
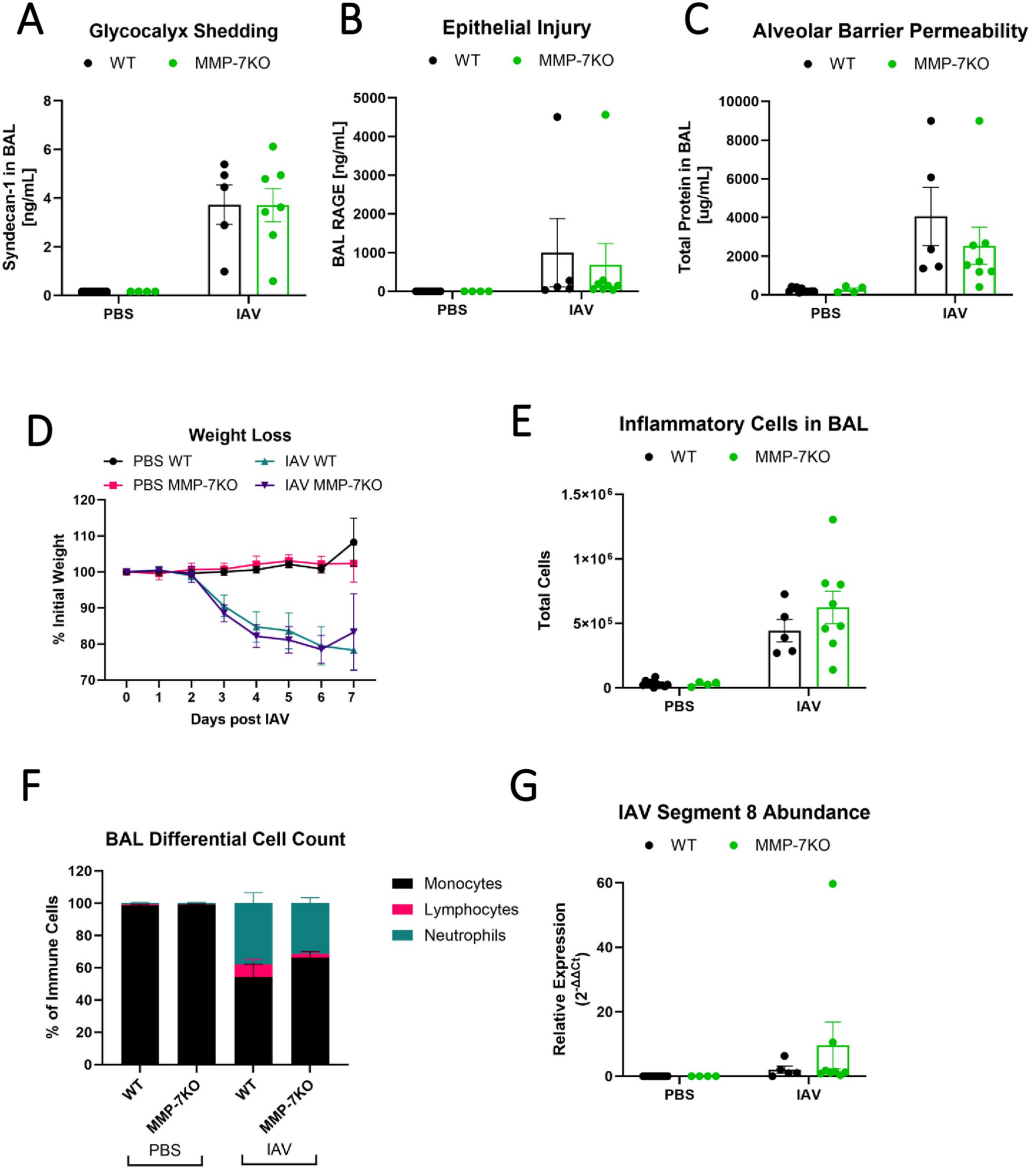
MMP-7 is not required for glycocalyx shedding, barrier permeability, or inflammation during IAV infection. MMP-7KO or WT littermate control C57BL/6 were intranasally administered PR8 influenza or PBS and monitored for up to 7 days. MMP-7KO mice had no significant difference in (A) glycocalyx shedding (n = 4–11 [5 male, 6 female]; two-way ANOVA), (B) BAL protein content (n = 4–11 [5 male, 6 female], two-way ANOVA), or (C) weight loss compared to WT controls (n = 4–11 [5 male, 6 female], mixed model ANOVA). MMP-7KO mice did not have significantly altered (D) number or (E) composition of inflammatory cells present in the lung on day 6. (n = 4–11 [5 male, 6 female], two-way ANOVA). (F) MMP-7KO mice have similar viral load to WT littermates as seen by ΔΔCt analysis to same day WT control (n = 4–11 [5 male, 6 female], two-way ANOVA). There are no statistically significant sex-specific differences in these parameters.

To determine the functional role of MMP-7 during IAV infection, we infected MMP-7 null mice (MMP-7KO) with IAV and examined glycocalyx shedding and lung injury on day 7 post-infection. MMP-7KO mice had no significant differences in glycocalyx shedding (Fig 6A), epithelial injury (Fig 6B), alveolar-capillary barrier permeability (Fig 6C), or weight loss (Fig 6D) during IAV compared to WT littermate controls. MMP-7KO mice had similar BAL inflammation as WT controls (Fig 6E and 6F). There was also no difference in IAV viral load (Fig 6G). Taken together, these data suggest that MMP-7 is not required for IAV-associated glycocalyx shedding, permeability, or inflammation.

## Discussion

In this manuscript, we use the pan-MMP inhibitor Ilomastat to show that MMPs are the sheddases associated with cleavage of the alveolar epithelial glycocalyx during IAV infection. We demonstrate that this process is independent of changes in inflammation, but that glycocalyx cleavage facilitates increased viral load. We also show that pan-MMP inhibition reduces epithelial injury, in addition to systemic injury as measured by weight loss. Our data demonstrate that MMP-7 is highly expressed in IAV-infected lung epithelial cells, whereas its inhibitor TIMP-1 is expressed in adjacent non-infected cells. However, global deletion of MMP-7 was not sufficient to alter glycocalyx cleavage, barrier permeability, or lung inflammation. These findings support the concept that IAV infection increases MMPs, leading to glycocalyx shedding, and increased lung injury.

These studies provide insight into the molecular mechanisms driving glycocalyx shedding during IAV and builds on previous work observations that IAV can destroy the alveolar epithelial glycocalyx [10, 18, 29]. We show that the pharmacologic inhibition of MMPs reduced glycocalyx shedding and that there is differential spatiotemporal regulation of MMP-7 and its inhibitor TIMP-1. These findings are consistent with previous studies showing that the specific MMP expressed varies depending on the injuring stimulus. In murine models of direct lung injury, IT LPS upregulates MMP-9 but not MMP-2 [6], whereas bleomycin upregulates MMP-2 but not MMP-9 [8]. MMP-7 cleaves the glycocalyx anchoring protein Syndecan-1 during *Pseudomonas aeruginosa* infection [15]. In contrast, IAV upregulates MMP-9 on neutrophils [17] and MMP-14 on myeloid cells [18], contributing to inflammation and extracellular matrix destruction, respectively. Our previously published bulk RNA sequencing also found that IAV significantly upregulated MMP-7, MMP-8, MMP-10, MMP-13, MMP-14, and MMP-25 [10]. Our current data add to this information not only by establishing the causal nature of MMPs and glycocalyx shedding using the inhibitor ILO but by examining the spatial and temporal relationships between IAV infection, MMP-7, and TIMP-1. The important cell-specific spatial relationships likely explain differences in cell-specific RNA expression (seen using RNAScope) and total lung protein levels (assessed by ELISA). Overall, our data suggest a working model in which IAV increases MMP-7 (or other as-yet-unknown sheddases) to cleave the glycocalyx and augment infection, then the lung upregulates TIMP-1 in nearby cells to control MMP expression locally, preventing further glycocalyx shedding, thereby attenuating infection and limiting the area of lung injury.

Our data also support a growing body of work showing that glycocalyx shedding is associated with more severe illness in both animal models and human studies. During human IAV infection, glycocalyx fragments in the serum are linked to poor prognosis of IAV disease [30]. The amount of glycocalyx shedding in the airspace of humans with direct lung injury (usually due to pneumonia) has a predictive value for estimating the severity and duration of ARDS [11]. Furthermore, loss of syndecan-1 has been linked to more severe acute lung injury in mice as intact syndecan-1 suppresses bronchial epithelial apoptosis after IAV [29]. Taken together with these studies, our data builds on the idea that destruction of the lung epithelial glycocalyx is a significant contributor to lung epithelial injury during viral infection.

Our new data support a key role for glycocalyx shedding in modulating IAV burden in the airspace. We found that inhibition of glycocalyx shedding by Ilomastat in vivo attenuates IAV load and that artificially inducing glycocalyx shedding on epithelial cells in vitro promotes increased viral load. When considering potential mechanisms for this, it should be noted that glycocalyx shedding results in two phenotypes: 1) increased syndecan and other glycocalyx fragments in BAL and 2) changes to the extracellular matrix remaining on the epithelial layer. Which aspect of glycocalyx cleavage affects IAV virulence is not well understood. Previous reports have found that sulfated derivatives of chitooligosaccharides administered orally reduce pulmonary titers of IAV by interacting with hemagglutinin on the virion’s surface [31]. The glycosaminoglycan fragments produced by IAV-induced viral shedding are also highly sulfated [10], thus may also impact the ability of IAV to propagate between cells. However, it is currently unknown if the shed glycocalyx fragments can directly bind the IAV virion or whether removal of the glycocalyx from the cell surface facilitates viral propagation by increasing access to the epithelial cells. It is also possible that cleavage of the glycocalyx exposes more of the sialic acid receptors in the extracellular matrix that are needed for IAV cellular entry [32] and that inhibition of shedding may sterically block receptor access. Further experimentation is needed to determine the driving force behind the antiviral phenotype seen in our data.

We found that broad MMP inhibition with Ilomastat affected glycocalyx shedding and viral dynamics. However, because the effects of ILO were distinct from genetic deletion of MMP-7, it is likely that other MMPs may have important roles in glycocalyx shedding during IAV infection. This is consistent with our prior work that reported upregulation of multiple MMPs during experimental IAV infection [10]. In particular, MMP-14 is a possible candidate as one study found MMP-14 on myeloid cells to be important in extracellular matrix damage during IAV infection, though this work did not specifically test for effects on syndecan-1 or the glycocalyx [18]. There is *in vivo* redundancy among MMPs as MMPs have shared substrates [33] so there may be compensation in single knock out models. Previous reports have found this to be true, with MMP-9KO animals having higher MMP-2 expression than wild-type littermates upon LPS stimulation [6]. Future studies could include assessment of influenza pathogenesis in other MMP transgenic mice, either alone or in combination or could use conditional or cell-specific knockout mice to dissect the complex roles of MMPs in IAV infection. Furthermore, it is also possible that MMP-7 is not responsible for glycocalyx shedding directly but rather activates another MMP that subsequently cleaves the glycocalyx, in a manner similar to how MMP-14 activates MMP-2 [34, 35]. Another possibility is that MMP-7 upregulation is a result of glycocalyx shedding, rather than the cause. Highly sulfated glycosaminoglycans similar to those released during IAV-associated glycocalyx shedding can boost the activity and alter the substrate specificity of MMP-7 [36]. Due to the broad nature of pan-MMP inhibition, it is also possible that ILO has off target effects that either drive cellular changes or inhibit additional proteases, potentially reducing glycocalyx shedding independent of MMPs. Ultimately, more research is needed to determine the specific role of MMP-7 in glycocalyx shedding by IAV and what other proteases might be responsible for glycocalyx shedding in this context.

In conclusion, we show that MMPs are responsible for glycocalyx shedding during IAV. MMP-7 and its inhibitor TIMP-1 are upregulated after IAV infection and have distinct localization patterns within the lung. We also show a novel role of glycocalyx shedding in modulation of viral dynamics. Overall, our study adds to a growing body of literature showing that the alveolar epithelial glycocalyx plays an important role in IAV infection in the lung.

## Supporting information

S1 Fig Expression of MMP-7, TIMP-1, MMP-2, MMP-9, and TIMP-2 during IAV infection. RNA expression in whole lung lysates of mice given PBS or IAV was measured over time for (A) MMP-2 (overall p = 0.005 for infection, overall p = 0.227 for time), (B) MMP-9 (overall p = 0.234 for infection, overall p = 0.0002 for time), and (C) their inhibitor TIMP-2 (overall p = 0.896 for infection, overall p = 0.399 for time). n = 9–11 [9 male, 4 female], two-way ANOVA for each with significant post hoc relationships shown.
(PDF)

S2 Fig MMP-7 colocalizes with IAV-infected cells and TIMP-1 is adjacent to infected cells. Low magnification imaging of IAV genome segment 8 and (A, B) MMP-7 or (C, D) TIMP-1 localization in lung slices visualized by in situ RNA hybridization. 10x fields representative of IAV and PBS control mice shown at day 3 and day 7.
(PDF)

S3 Fig MMP-2, MMP-9, and TIMP-2 RNA do not co-localize with IAV. Low magnification imaging of IAV genome segment 8 and (A, B) MMP-2, (C, D) MMP-9, or (E, F) TIMP-2 localization in lung tissue visualized by in situ RNA hybridization. 10x fields representative of infected and uninfected mice shown at day 3 and day 7.
(PDF)
