# Supplementary figures and images for: Matrix metalloproteinases mediate influenza A-associated shedding of the alveolar epithelial glycocalyx
Source: PLoS One. 2024 Sep 23;19(9):e0308648. doi: 10.1371/journal.pone.0308648 (PMC11419339; doi:10.1371/journal.pone.0308648)

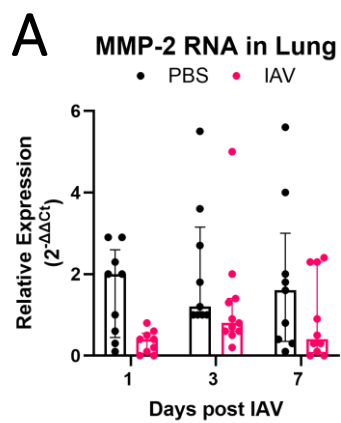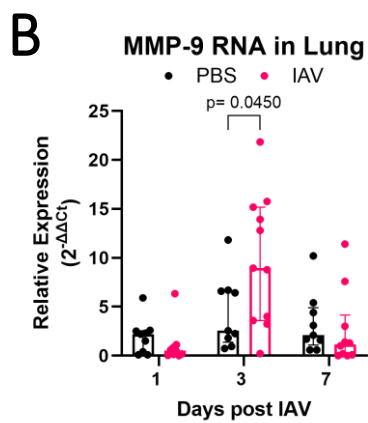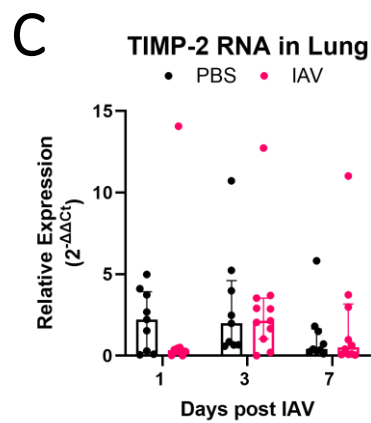

Supplement: S1 Fig — RNA expression in whole lung lysates of mice given PBS or IAV was measured over time for (A) MMP-2 (overall p = 0.005 for infection, overall p = 0.227 for time), (B) MMP-9 (overall p = 0.234 for infection, overall p = 0.0002 for time), and (C) their inhibitor TIMP-2 (overall p = 0.896 for infection, overall p = 0.399 for time). n = 9–11 [9 male, 4 female], two-way ANOVA for each with significant post hoc relationships shown. (PDF) [file pone.0308648.s001.pdf]

**A**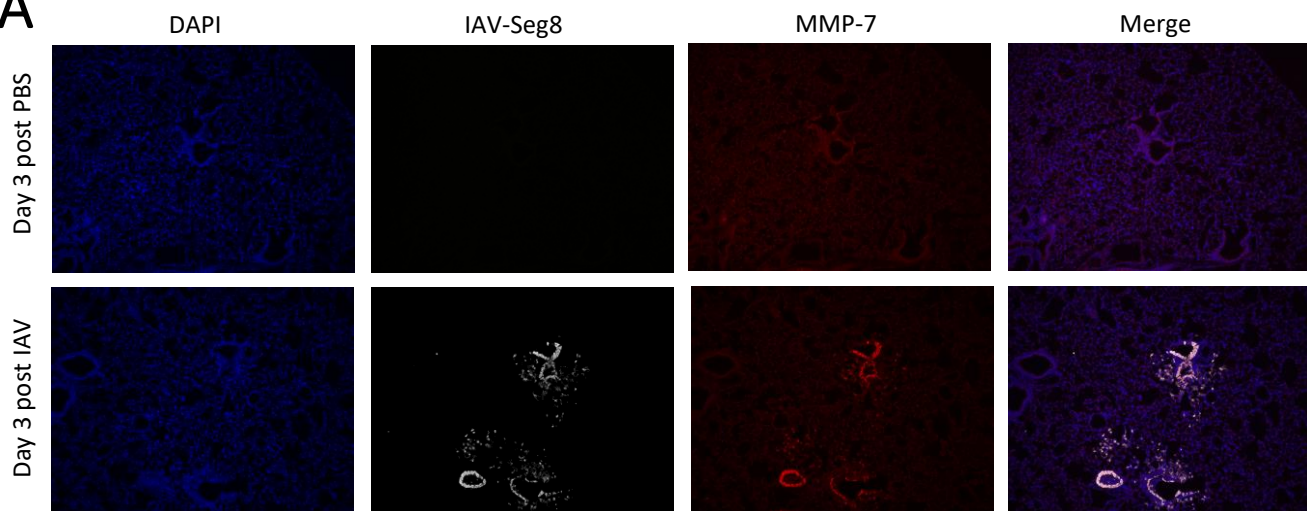**B**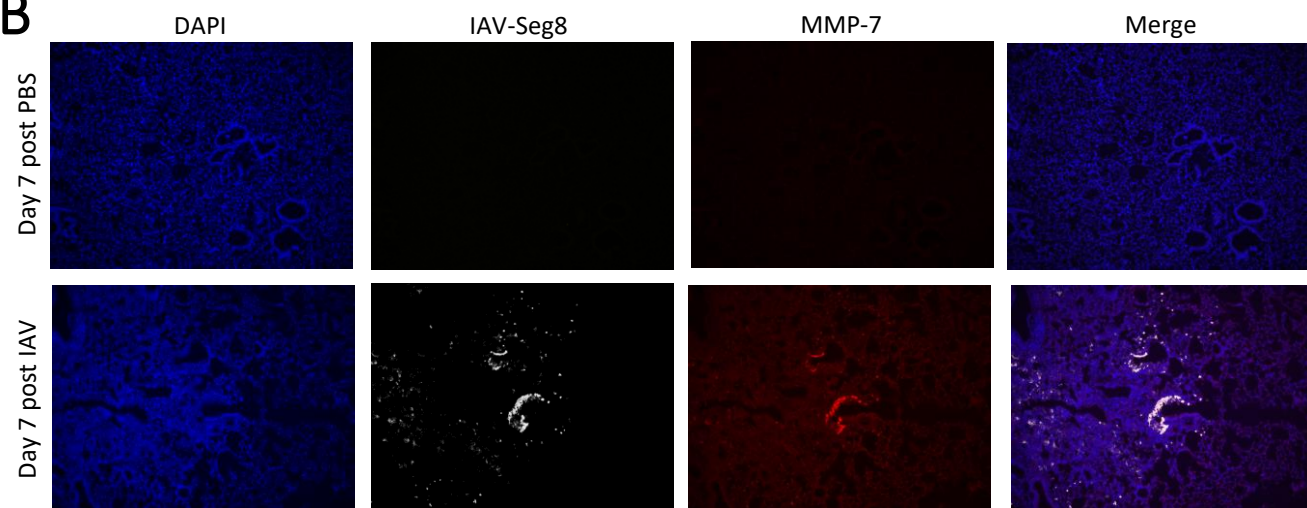

**C**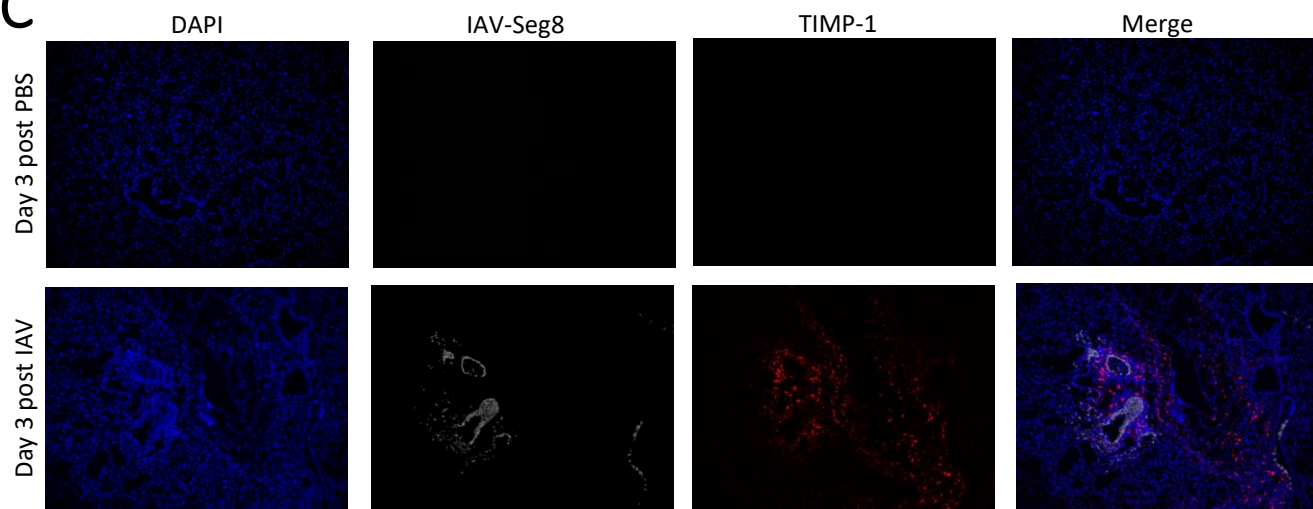**D**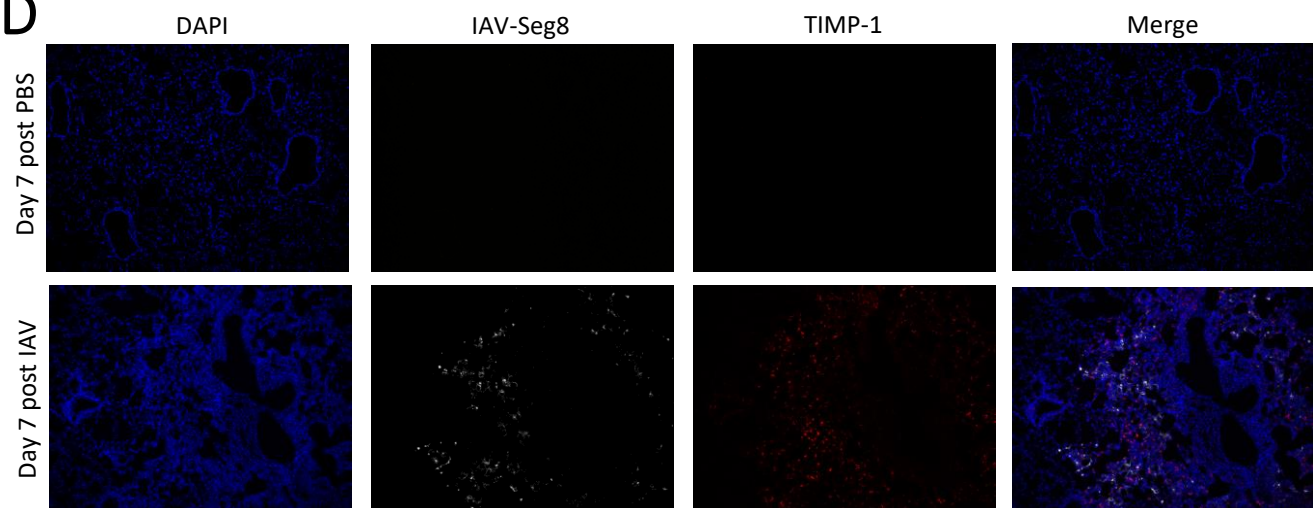

Supplement: S2 Fig — Low magnification imaging of IAV genome segment 8 and (A, B) MMP-7 or (C, D) TIMP-1 localization in lung slices visualized by in situ RNA hybridization. 10x fields representative of IAV and PBS control mice shown at day 3 and day 7. (PDF) [file pone.0308648.s002.pdf]

**A**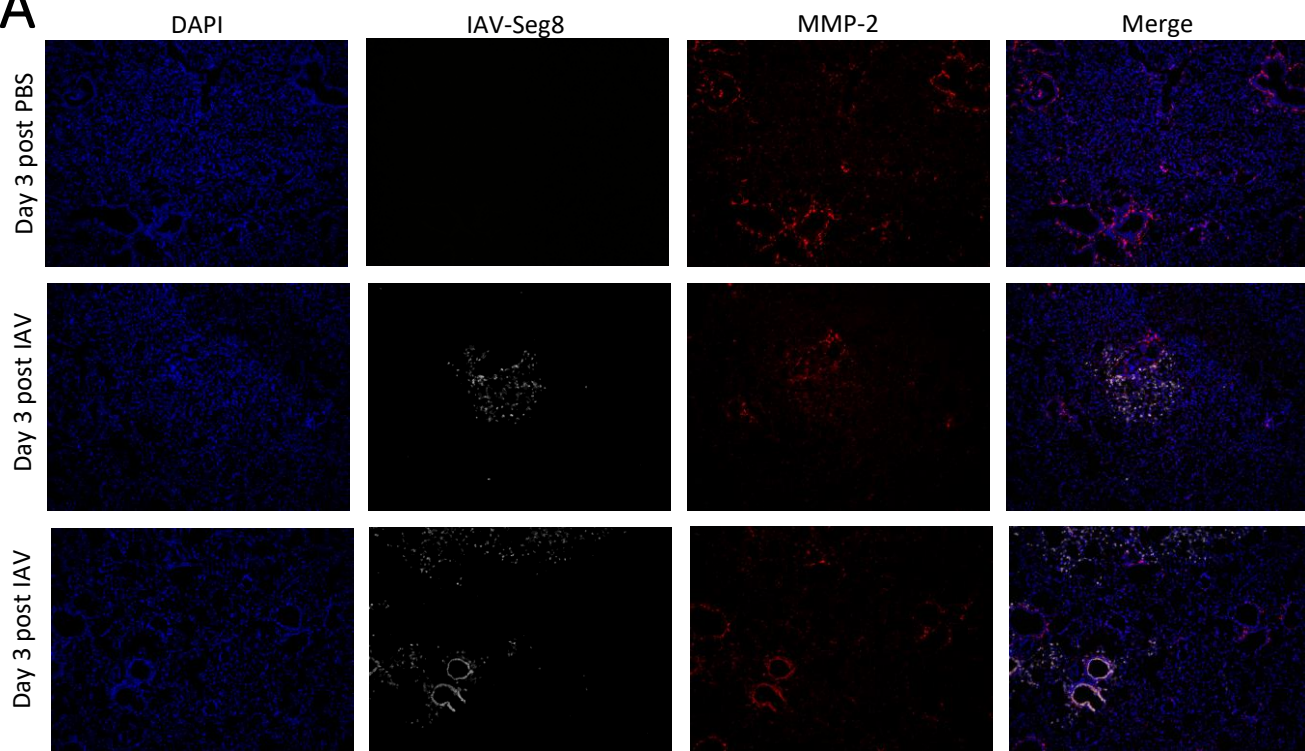**B**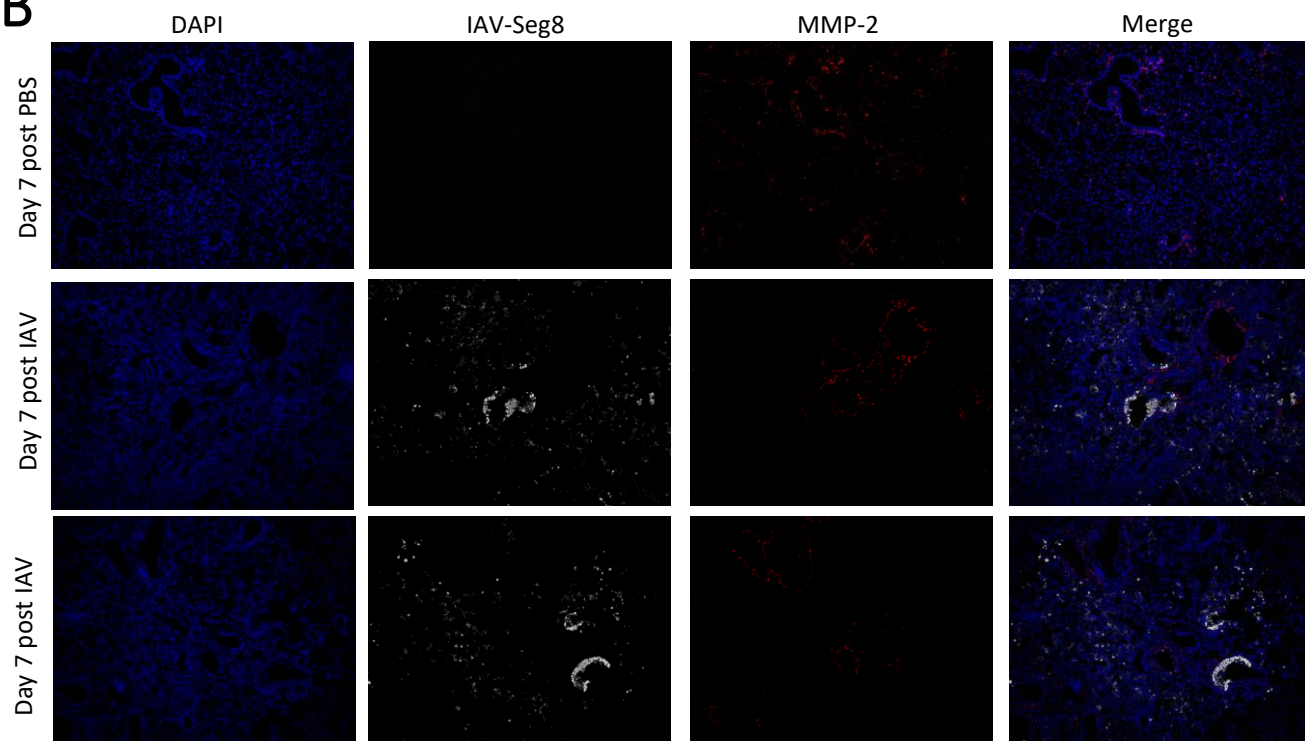

**C**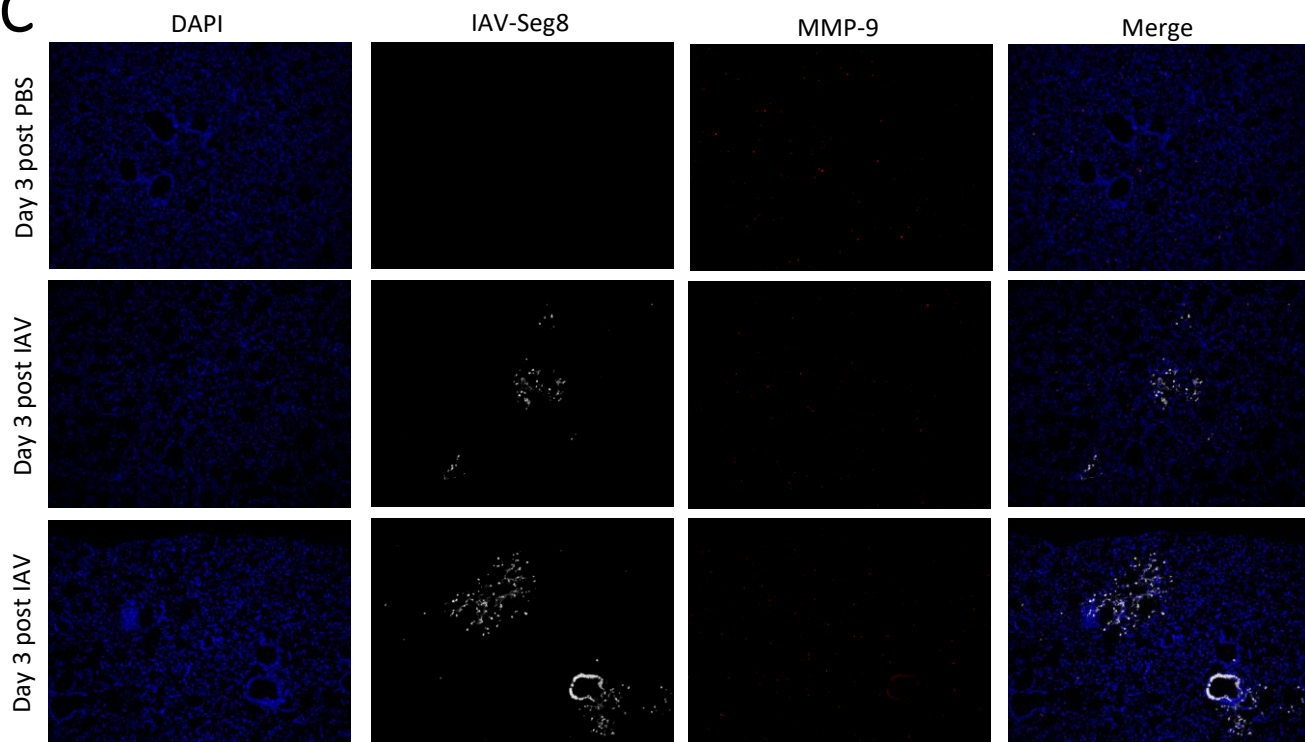**D**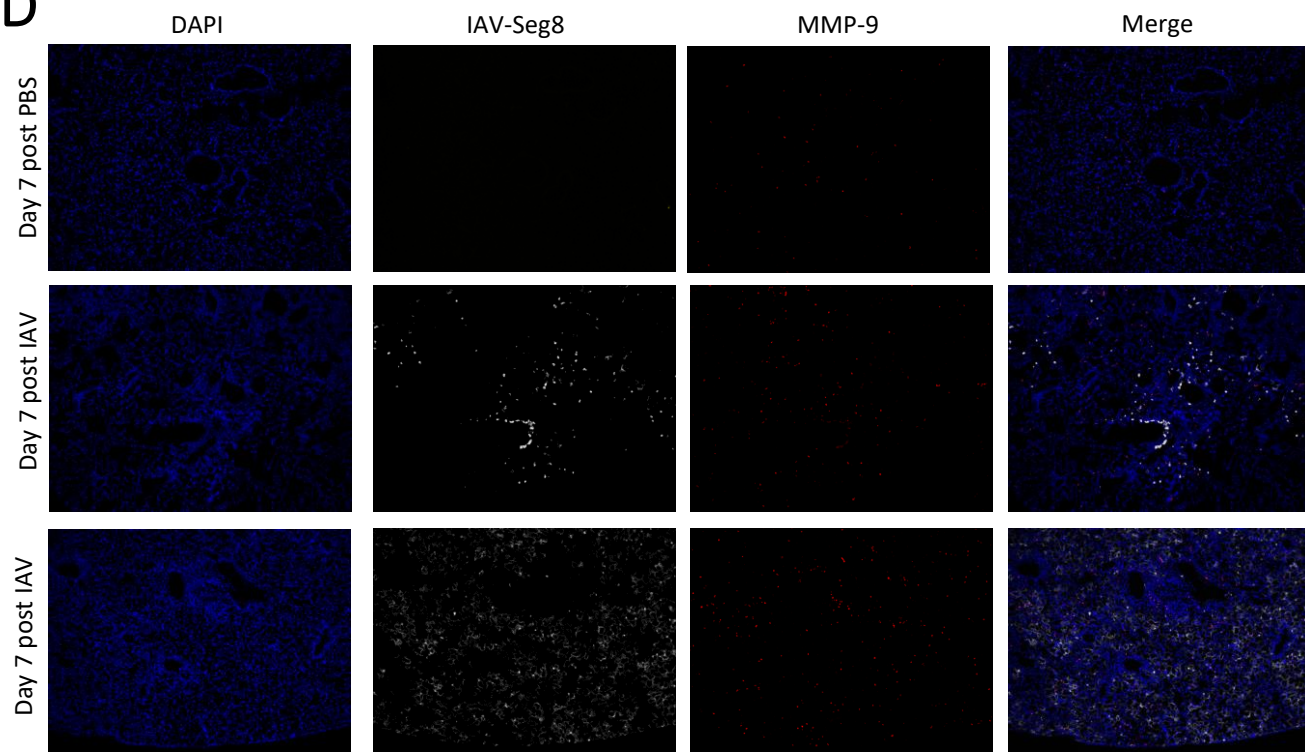

**E**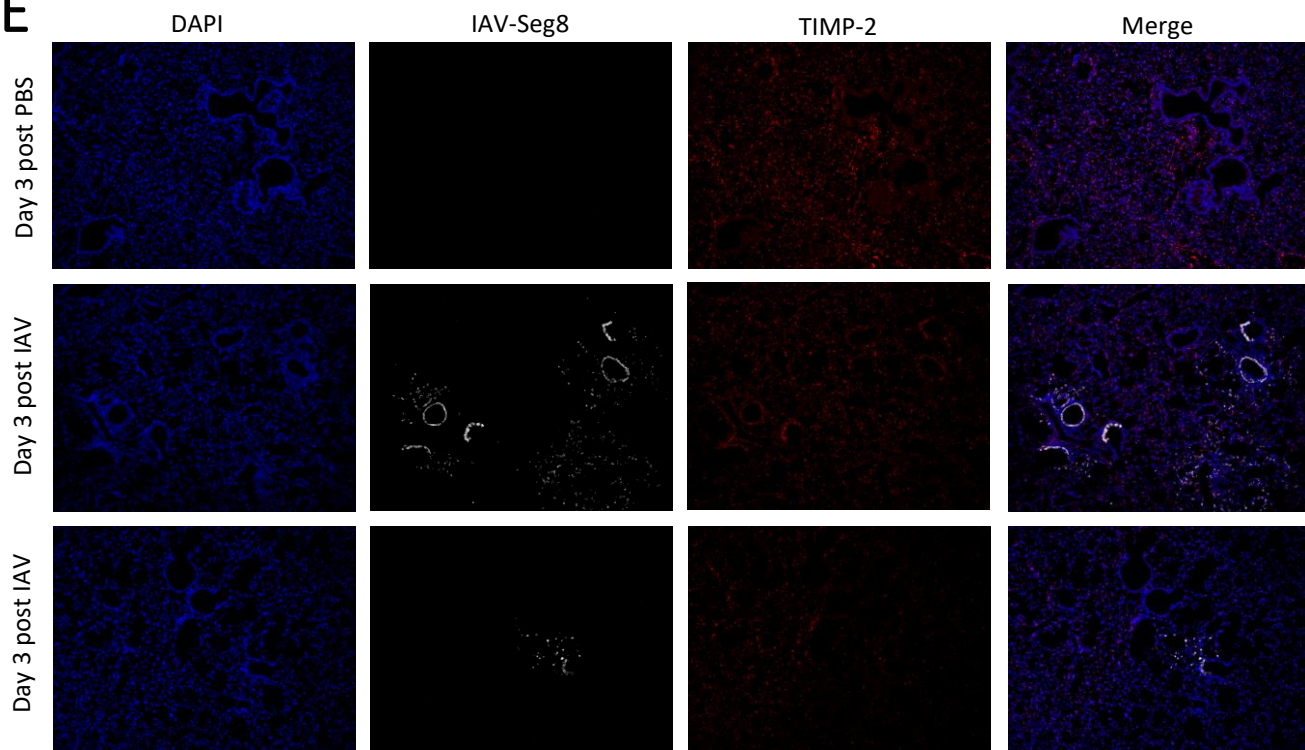**F**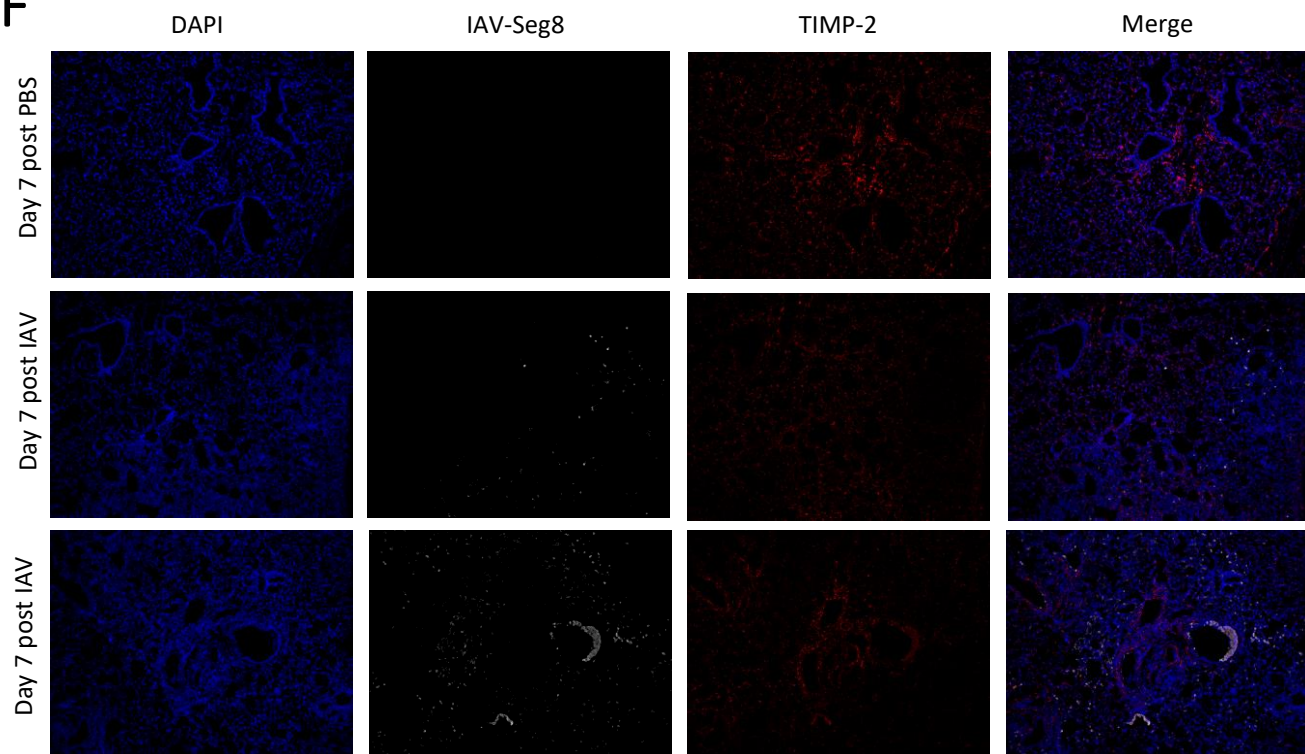

Supplement: S3 Fig — Low magnification imaging of IAV genome segment 8 and (A, B) MMP-2, (C, D) MMP-9, or (E, F) TIMP-2 localization in lung tissue visualized by in situ RNA hybridization. 10x fields representative of infected and uninfected mice shown at day 3 and day 7. (PDF) [file pone.0308648.s003.pdf]
